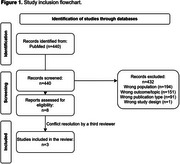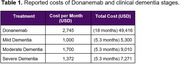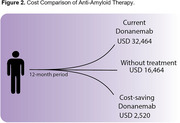# Cost‐saving analysis for adopting Anti‐Amyloid Therapies for Alzheimer's Disease

**DOI:** 10.1002/alz70860_106253

**Published:** 2025-12-23

**Authors:** Rhaná Carolina Santos, Julia Reis Mathias, Sonia Maria Dozzi Brucki, Ana Paula Beck da Silva Etges, Pedro Rosa‐Neto, Eduardo R. Zimmer, Wyllians Vendramini Borelli

**Affiliations:** ^1^ Federal University of Rio Grande do Sul, Porto Alegre, Rio Grande do Sul, Brazil; ^2^ Universidade do Vale do Rio dos Sinos, São Leopoldo, Rio Grande do Sul, Brazil; ^3^ Federal University of Rio Grande do Sul, PORTO ALEGRE, Rio Grande do Sul, Brazil; ^4^ Cognitive and Behavioral Neurology Unit, Medical School, University of São Paulo, São Paulo, Brazil; ^5^ Hospital das Clinicas HCFMUSP, Faculdade de Medicina, Universidade de Sao Paulo, Sao Paulo, Sao Paulo, Brazil; ^6^ Cognitive and Behavioral Neurology Unit, Hospital das Clinicas HCFMUSP, Faculdade de Medicina, Universidade de Sao Paulo, Sao Paulo, Sao Paulo, Brazil; ^7^ McConnell Brain Imaging Centre, Montreal Neurological Institute, McGill University, Montreal, QC, Canada; ^8^ Montreal Neurological Institute, Montreal, QC, Canada; ^9^ Universidade Federal do Rio Grande do Sul, Porto Alegre, RS, Brazil; ^10^ McGill University, Montreal, QC, Canada; ^11^ Brain Institute of Rio Grande do Sul ‐ Pontifícia Universidade Católica do Rio Grande do Sul, Porto Alegre, Rio Grande do Sul, Brazil; ^12^ Universidade Federal do Rio Grande do Sul, Porto Alegre, Rio Grande do Sul, Brazil; ^13^ Centro de Memória, Hospital Moinhos de Vento, Porto Alegre, RS, Brazil; ^14^ Clinical Hospital of Porto Alegre, Porto Alegre, Rio Grande do Sul, Brazil; ^15^ Brain Institute of Rio Grande do Sul (InsCer), PUCRS, Porto Alegre, Rio Grande do Sul, Brazil

## Abstract

**Background:**

Anti‐amyloid drugs hold promise in delaying clinical stages of dementia, though they may be rarely used in Low and Middle‐Income Countries (LMIC) due to treatment costs. Herein, we aimed to evaluate the potential cost‐savings associated with the use of Donanemab for Alzheimer's Disease (AD) in the perspective of the Brazilian healthcare system.

**Method:**

We conducted a systematic review of studies estimating the costs of dementia stages in Brazil between 1997 and 2024. Key terms included costs and cost analyses; Alzheimer's Disease; Dementia; and Brazil. Costs of Donanemab were sourced from industry reports, and a cost‐saving analysis compared healthcare costs for managing mild and moderate AD. The treatment's efficacy was evaluated based on performance in the Clinical Dementia Rating scale, considering that the treatment is able to delay the progression between mild and moderate dementia stages by an average of 5.3 months.

**Result:**

Three studies evaluating costs of dementia in Brazil were retrieved in the systematic review (Figure 1), with a cost associated with dementia ranging from USD 536.27 to USD 1,376.28. Monthly costs for mild, moderate, and severe dementia stages were estimated at USD 1,000, USD 1,700, and USD 1,372, respectively (Table 1). The cost of Donanemab treatment (18 months) was estimated at USD 48,696, along with approximately USD 720 for the six magnetic resonance imaging exams required during the treatment period. Reducing progression from a patient with mild to moderate dementia stage for 5.3 additional months can reduce the cost of approximately USD 3,710 (USD 700 per month). For the total 18‐month treatment period, the cost saving threshold to make anti‐amyloid economically viable in Brazil would be estimated around USD 210 monthly, or a total cost of USD 3,780.

**Conclusion:**

Anti‐amyloid treatment in Brazil can collaborate to reduce Dementia costs in Brazil and, potentially, become a cost‐effective strategy. Transposing the results presented here to the estimated number of AD patients in Brazil of 1.2 million people emphasizes the potential economic implications in terms of public policies and the healthcare system as a whole.